# Mechanisms of the NAD^+^ salvage pathway in enhancing skeletal muscle function

**DOI:** 10.3389/fcell.2024.1464815

**Published:** 2024-09-20

**Authors:** Mengzhu Su, Fanghui Qiu, Yansong Li, Tongtong Che, Ningning Li, Shuangshuang Zhang

**Affiliations:** ^1^ Cancer Institute, The Affiliated Hospital of Qingdao University, Qingdao University, Qingdao Cancer Institute, Qingdao, China; ^2^ School of Physical Education, Qingdao University, Qingdao, China

**Keywords:** NAD^+^, NAMPT, skelelal muscle, aging, T2DM

## Abstract

Nicotinamide adenine dinucleotide (NAD^+^) is crucial for cellular energy production, serving as a coenzyme in oxidation-reduction reactions. It also supports enzymes involved in processes such as DNA repair, aging, and immune responses. Lower NAD^+^ levels have been associated with various diseases, highlighting the importance of replenishing NAD+. Nicotinamide phosphoribosyltransferase (NAMPT) plays a critical role in the NAD^+^ salvage pathway, which helps sustain NAD^+^ levels, particularly in high-energy tissues like skeletal muscle.This review explores how the NAMPT-driven NAD^+^ salvage pathway influences skeletal muscle health and functionality in aging, type 2 diabetes mellitus (T2DM), and skeletal muscle injury. The review offers insights into enhancing the salvage pathway through exercise and NAD^+^ boosters as strategies to improve muscle performance. The findings suggest significant potential for using this pathway in the diagnosis, monitoring, and treatment of skeletal muscle conditions.

## 1 Introduction

**FIGURE 1:**
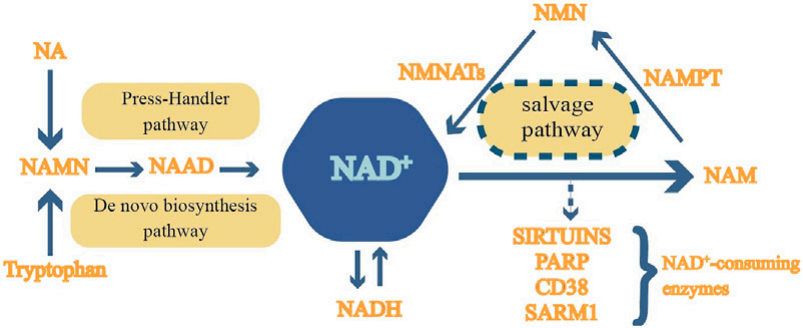
Mammalian NAD^+^ metabolism.

Nicotinamide phosphoribosyltransferase (NAMPT) was first identified in B cells, where it helps mature B cells, earning it the initial name Pre-B cell colony-enhancing factor (PBEF) (Samal et al., 1994). Additionally, NAMPT, known as “Visfatin” (Fukuhara et al., 2005) for its insulin-like effects, is secreted by visceral fat. Analysis of the tissue origins of 719 cDNA clones revealed that NAMPT is expressed in nearly all organs, tissues, and cells (Friebe et al., 2011), indicating that this protein may have multiple regulatory functions in human physiological processes. NAD^+^ is a coenzyme essential for redox reactions in metabolic processes, including glycolysis, the citric acid cycle, and oxidative phosphorylation (Houtkooper et al., 2010). It also acts as a substrate for various NAD^+^-dependent enzymes, influencing processes regulated by enzymes like silent information regulators (Sirtuins), poly (ADP-ribose)polymerases (PARPs), cyclic ADP-ribose (cADPR) and sterile alpha and TIR motif-containing 1(SARM1)synthases (Cantó et al., 2013; Essuman et al., 2017; Malavasi et al., 2008), thereby impacting cellular functions like DNA repair and signaling. NAD^+^ is replenished through the *De novo* biosynthesis pathway, Preiss-Handler pathway and salvage pathway, with 85% of total NAD^+^ being produced by the salvage pathway. Revollo et al. (2007). In the salvage pathway, NAMPT plays a central role by converting nicotinamide (NAM) into nicotinamide mononucleotide (NMN), which is then converted into NAD^+^ by enzymes NMNAT1-3, completing the cycle of NAD^+^ synthesis and breakdown (Revollo et al., 2004) which is depicted in Figure 1.

During intense physical activity, skeletal muscles rely heavily on aerobic oxidation in mitochondria for energy. The tricarboxylic acid cycle, integral to this process, involves NAD^+^ in three out of four dehydrogenation reactions. Reduced NAD^+^ levels can impair muscle contractions and disrupt mitochondrial energy metabolism, potentially triggering an energy crisis in skeletal muscles. NAMPT-driven NAD^+^ salvage pathway is critical for maintaining NAD^+^ levels in skeletal muscles. Eliminating NAMPT in the skeletal muscles of adult mice led to an 85% reduction in NAD^+^ content (Frederick et al., 2016). Basse et al. (2021) specifically knocked out NAMPT in the skeletal muscle of fetal mice, the mice began to exhibit abnormal muscle development, impaired function, and decreased motor ability from the age of 4 weeks, gradually progressing to severe myopathy. Despite a normal muscle stem cell pool, it did not alleviate the progression of the myopathy. Further research by Frederick and team shows that mice lacking NAMPT are smaller and exhibit reduced muscle mass as they age, a condition linked to increased expression of genes related to muscle damage and inflammation, as well as decreased metabolic activity (Frederick et al., 2016). Six weeks after knocking out NAMPT, three-month-old male mice exhibited significant neurite loss in both motor and hippocampal neurons (Wang et al., 2017). This disruption of neural regulatory mechanisms, essential for governing muscular activity, led to abnormal movement, muscle atrophy, and ultimately, the death of the mice (Shen et al., 2023); NAD^+^ supplementation has been shown to improve pathological changes in rats with muscular dystrophy through several mechanisms: enhancing mitochondrial function, increasing the expression of structural proteins like α-dystrobrevin, decreasing poly (ADP-ribosyl)ation, and reducing inflammation and fibrosis, underscoring its importance in muscle health and function (Ryu et al., 2016). Disruptions in NAMPT homeostasis impair the ion transport function of the mitochondrial permeability transition pore (mPTP) (Briston et al., 2019). When NAMPT levels are deficient or insufficient, the mPTP stays persistently open, making mitochondria more sensitive to Ca^2+^ (Basse et al., 2021). This sensitivity causes mitochondrial Ca^2+^ overload, triggering oxidative stress in skeletal muscle and further opening the mPTP. This vicious cycle leads to mitochondrial swelling and a decrease in membrane potential. NAMPT deficiencies in skeletal muscle significantly reduce mitochondrial fusion proteins (Mfns) expression and increase mitochondrial fission protein (Drp1) levels and protein acetylation. This imbalance leads to excessive mitochondrial fission and altered post-translational modifications, disrupting mitochondrial structure (Kim et al., 2014). As a result, vital components such as cardiolipin, phosphatidylglycerol, enzymes of the electron transport chain, and citrate are markedly reduced, impairing mitochondrial function and biogenesis (Agerholm et al., 2018). These structural and functional mitochondrial abnormalities lead to oxidative stress and inflammation, which accelerate protein degradation, impair regenerative capacity, and exacerbate skeletal muscle dysfunction (Lee et al., 1998; Wanagat et al., 2001). In conclusion, NAMPT plays a vital role in regulating skeletal muscle growth, development, and contraction by influencing mitochondrial structure and function via its involvement in NAD⁺ synthesis. Under pathological conditions, the enhancement of NAD⁺-centered metabolic pathways has been shown to significantly enhance skeletal muscle function. Subsequent sections will explore how the NAD⁺ salvage pathway mitigates issues such as aging, type 2 diabetes mellitus (T2DM), and skeletal muscle injuries.

## 2 NAD^+^ salvage pathway improves age-related decline in skeletal muscle function

### 2.1 Possible physiological mechanisms underlying the development of sarcopenia

The Asian Working Group for Sarcopenia defines sarcopenia as an age-related condition marked by a decline in muscle mass, strength, and physical function (Chen et al., 2014). Oxidative stress occurs when an imbalance between oxidants and antioxidants leads to excessive oxidative activity, damaging vital molecules such as sugars, lipids, proteins, and DNA (Sies et al., 2017). Aging is associated with a decrease in the quantity and activity of antioxidant enzymes, fostering an overproduction of reactive oxygen species (ROS), which disrupt cellular redox balance (Sullivan-Gunn and Lewandowski, 2013). Research has conclusively demonstrated that oxidative stress significantly contributes to sarcopenia (Coto-Montes et al., 2017) by accelerating muscle protein degradation through hyperactivation of the ubiquitin-proteasome and autophagy-lysosome pathways, while simultaneously impeding protein synthesis (Jang et al., 2020). Furthermore, oxidative stress impairs mitochondrial biogenesis, diminishes ATP production, and disrupts mitochondrial autophagy, leading to energy metabolism disorders in skeletal muscle fibers and exacerbating muscle wasting (Jang et al., 2010). In addition, oxidative stress stimulates the accumulation of inflammatory cytokines and enhances the phagocytic activity of macrophages and neutrophils, triggering a chronic inflammatory response that intensifies oxidative stress (Bartnik et al., 2000.). This vicious cycle further damages muscle fiber structure and function (Chen et al., 2022). Mitochondrial dysfunction, central to aging (López-Otín et al., 2023), involves the accumulation of mtDNA mutations, imbalances in protein synthesis and degradation, instability of respiratory chain complexes, and altered mitochondrial dynamics. These changes undermine mitochondrial integrity and function, contributing to muscle fiber degeneration. Deterioration in mitochondrial function increases ROS production and mitochondrial membrane permeability, precipitating inflammation and cellular destruction (Hepple, 2014).

In the muscles of aged mice and elderly humans, levels of autophagy markers, including autophagy-related protein7 (ATG7)and Light Chain 3 (LC3) lipidation, are reduced, indicating impaired autophagic function (Carnio et al., 2014). Skeletal muscles in ATG7-specific knockout mice exhibit muscle atrophy, reduced strength, and pathological features characteristic of myopathy, with the decline in muscle strength becoming more pronounced with aging (Masiero et al., 2009). Impaired autophagy disrupts the protein quality control system, leading to increased protein aggregates that further exacerbate sarcopenia (Jiao and Demontis, 2017). Aging is often accompanied by chronic low-grade inflammation, primarily associated with senescent cells and their senescence-associated secretory phenotype (SASP). The SASP increases with age, releasing pro-inflammatory cytokines that contribute to both inflammation and aging (Fulop et al., 2018; Fulop et al., 2021). Previous studies have shown that elevated IL-6 levels can predict disability in the elderly, likely due to the direct effects of IL-6 on muscle atrophy (Ferrucci et al., 1999). Additionally, IL-1β levels are negatively correlated with walking speed, further linking inflammation to the decline in skeletal muscle function (Morawin et al., 2023). Inflammatory cytokines activate protein degradation and inhibit protein synthesis, leading to sarcopenia (Jo et al., 2012).

Age-related structural and functional decline of the neuromuscular junction (NMJ) significantly contributes to muscular dystrophy. Aging leads to the thinning and degradation of motor neuron axons, which exhibit disordered terminal branching (Padilla et al., 2021). Decreased reactivity of terminal Schwann cells and a reduction in the quantity and density of acetylcholine receptors on muscle fibers contribute to neural innervation failure and disrupted muscle contractions (Alice et al., 2016).Skeletal muscle stem cells (MuSCs) are crucial for the regenerative and repair functions of skeletal muscles due to their quantity and functionality (Jiang et al., 2024). In male rats between 15 and 18 months of age, the number of MuSCs declines with advancing age, and they undergo morphological changes, including flattening, enlargement, and a loss of three-dimensional structure. Consequently, these alterations weaken the proliferation and self-renewal potential of MuSCs, causing a shift from a reversible dormant state to a permanently quiescent state, which compromises the regenerative capacity of skeletal muscles (Pedro et al., 2021).

### 2.2 The NAD^+^ salvage pathway has been shown to ameliorate sarcopenia and its underlying mechanisms

NAD^+^ was one of the most prominent metabolites that decreased in older adults, and this decline was even more pronounced in impaired older adults (Janssens et al., 2022). This outcome is a consequence of both reduced synthesis efficiency and increased consumption. Across various tissues in humans and mice, the level of NAMPT in the NAD^+^ salvage pathway declines with age (De Guia et al., 2019; Stein and Imai, 2014; Xing et al., 2019), resulting in decreased synthesis of NAD^+^. Furthermore, as the aging process progresses, the accumulation of DNA damage, inflammation, and oxidative stress intensifies, leading to an enhancement in the activity of NAD^+^ consuming enzymes such as CD38 and PARPs (Covarrubias et al., 2020; Goody and Henry, 2018; Khaidizar et al., 2021; Schultz and Sinclair, 2016; Silva et al., 2023). Enhancing NAD^+^ metabolism has emerged as a viable therapeutic approach to mitigate age-related conditions and extend lifespan, as supported by numerous studies (Gerdts et al., 2015; Krafczyk and Klotz, 2022; Mouchiroud et al., 2013; Tzameli, 2012). Lifelong overexpression of NAMPT can maintain NAD^+^ content and functionality in the skeletal muscle of aged mice (Frederick et al., 2016). We summarize the mechanisms by which the NAMPT salvage pathway improves aging skeletal muscle, including alleviating oxidative stress, maintaining the stability of the NAD^+^ pool, promoting autophagy, reducing chronic low-grade inflammation, enhancing the quantity and function of MuSCs, and improving the formation of neuromuscular junctions.

Increasing NAD^+^ enhances the function of silent information regulator factor (SIRT1) (Khaidizar et al., 2017), which activates the forkhead box proteins (FoxOs)pathway (Olmos et al., 2013), increases levels of antioxidant enzymes like Superoxide Dismutase 2 (SOD2), strengthens antioxidant defense mechanisms, reduces ROS (Zhang et al., 2023), mitigates oxidative stress damage, and therefore retards the aging process in skeletal muscle. Activated SIRT1 acts as an antioxidant by suppressing NF-κB expression while boosting levels of peroxisome proliferator-activated receptor γ coactivator 1α(PGC-1α) and nuclear factor erythroid 2-related factor 2 (Nrf2), which are key regulators of cellular defense against oxidative stress (Tzameli, 2012). The malate-aspartate shuttle (MAS), essential for the exchange between mitochondrial NAD^+^ and cytoplasmic NADH (Yang et al., 2007), stabilizes mitochondrial NAD^+^ during increased energy demands, facilitated by PGC-1α activation in skeletal muscle (Kang and Li Ji, 2012). The mitochondrial unfolded protein response (UPRmt) is crucial for maintaining mitochondrial function and homeostasis. Enhancing NAD^+^ in skeletal muscle models activates UPRmt, balancing mitochondrial protein systems and improving function (Mouchiroud et al., 2013). The NAD^+^-dependent enzyme SIRT1 deacetylates LC3 at K49 and K51 sites, enabling LC3 to interact with nucleoproteins and translocate to the cytoplasm, where it binds with autophagy factors such as ATG7, thereby promoting autophagy in skeletal muscle (Huang et al., 2015). Adenosine 5‘-monophosphate (AMP)-activated protein kinase (AMPK) is a key initiator of autophagy (Ge et al., 2022), and SIRT1 can activate AMPK (Gao et al., 2020; Ruderman et al., 2010). AMPK, in turn, activates Unc-51-like autophagy activating kinase 1 (ULK1) through phosphorylation. ULK1 then activates downstream molecules such as Bcl-2-interacting coiled-coil protein 1 (Beclin1) and ATGs, thereby promoting the autophagy process (Holczer et al., 2020). Studies have also shown that AMPK protects cells from oxidative stress-induced aging by increasing autophagic flux and enhancing NAD^+^ levels (Han et al., 2016). The NLR family pyrin domain containing 3(NLRP3) inflammasome is an intracellular signaling complex that produces pro-inflammatory cytokines, such as IL-1β. Reducing NLRP3 inflammasome expression can enhance muscle fiber size and contractility (McBride et al., 2017). SIRT2 inhibits NLRP3 inflammasome activity by deacetylating NLRP3, thereby reducing aging-associated chronic inflammation (He et al., 2020). Oral administration of nicotinamide riboside (NR) can increase skeletal muscle NAD^+^ levels and reduce circulating inflammatory cytokines in skeletal muscle (Elhassan et al., 2019). The regulation of other inflammatory factors by NAD^+^ to improve skeletal muscle function is discussed in the section on T2DM in this paper.

**FIGURE 2:**
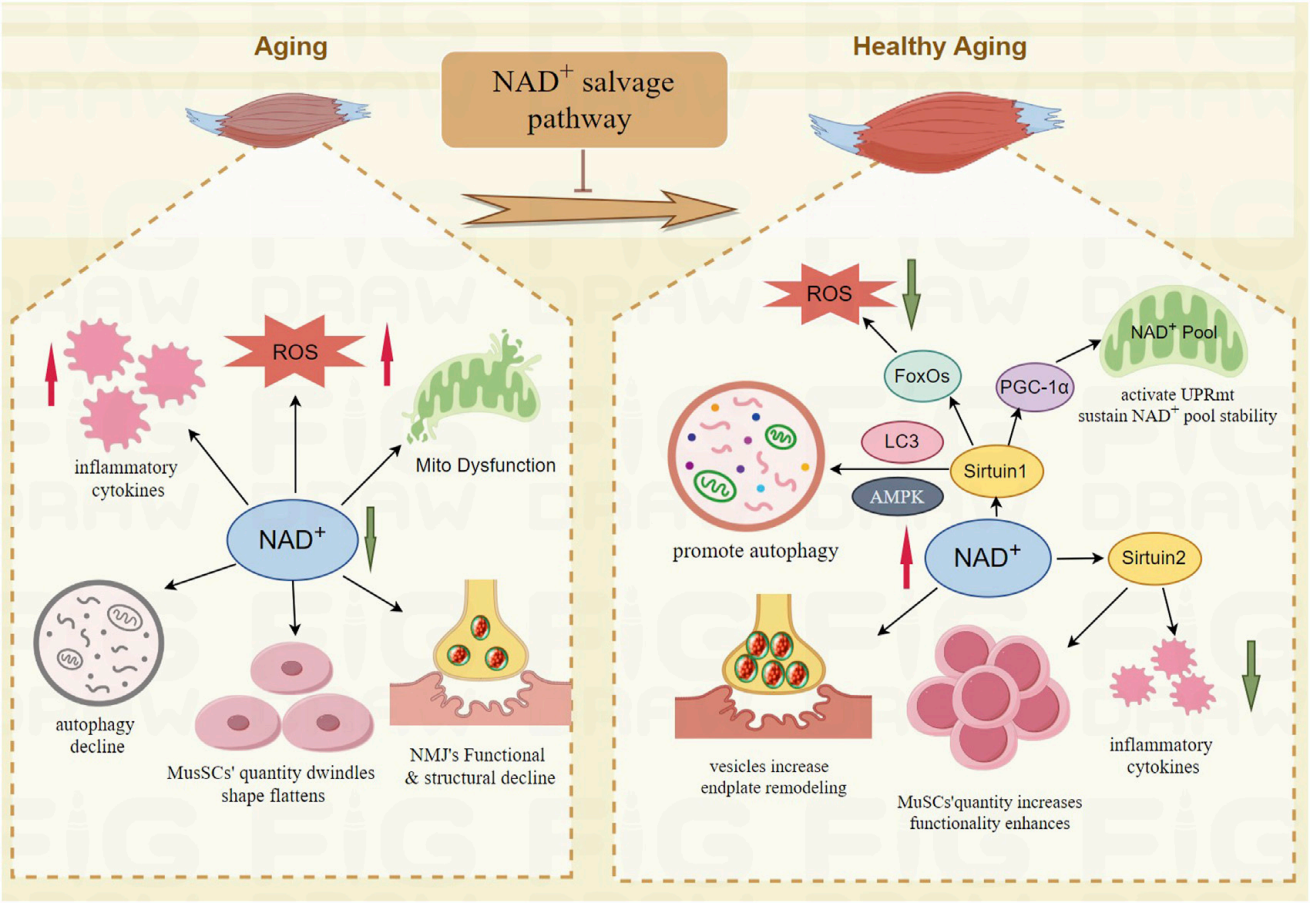
The mechanism of NAD^+^ salvage pathway improve the function of skeletal muscle in aging. NAD^+^-Sirtuin1- FoxOs mitigates oxidative stress damage; NAD^+^-Sirtuin1- PGC-1α improves the mitochondrial function; NAD^+^-Sirtuin1-AMPK promotes autophagy; NAD^+^-Sirtuin2 reduces chronic low-grade inflammation and enhances the quantity and functionality of MuSCs; NAD^+^ increases vesicles, remodels endplate of NMJ.

Research shows that the NAD^+^ salvage pathway enhances neuromuscular junctions (NMJ). Administering NMN to increase NAD^+^ levels improves vesicle count in axons, restores neuronal function, and maintains synaptic connectivity and transmission at NMJs (Oki et al., 2015; Samuel et al., 2020). Supplementing with NAMPT to increase NAD^+^ levels can boost MuSCs counts (Hongbo et al., 2016), as NAD^+^ promotes SIRT1 overexpression, which prevents aging of these cells (Ma et al., 2017), aiding in repair and maintaining the integrity of NMJs (Larouche et al., 2021). The TIR domain of SARM1 can cause axon damage, but overexpressing NAMPT in dorsal root ganglion neurons counters this effect, preventing axon degeneration and cell death (Gerdts et al., 2015). Overexpression of NAMPT also protects against glutamate excitotoxicity and neuronal apoptosis due to oxygen-glucose deprivation, thereby enhancing neuroprotection (Wang et al., 2016). Restoring the number and function of quiescent muscle stem cells in aging skeletal muscle helps improve sarcopenia. The NAD^+^-dependent SIRT1 deacetylates substrates such as histone H4K16ac, regulating gene expression and maintaining the quiescent state of stem cells (Ryall et al., 2015). NR supplementation can increase the number of MuSCs and enhance their self-renewal capacity (Hongbo et al., 2016). Paired Box 7(PAX7)is essential for maintaining the regenerative function of adult muscle stem cells (Sambasivan et al., 2011). SIRT2 deacetylates PAX7, promoting muscle stem cell self-renewal, inhibiting differentiation, and maintaining the undifferentiated state of MuSCs (Sincennes et al., 2021). Studies in twins have shown that NR promotes the activation of MuSCs from differentiation and fusion to formation of muscle fibers (Lapatto et al., 2023). In conclusion, the NAD^+^ salvage pathway holds therapeutic potential for treating sarcopenia by reversing pathogenic processes, as depicted in Figure 2.

## 3 NAD^+^ salvage pathway in improving T2DM skeletal muscle pathology

### 3.1 Decreased skeletal muscle contractile function in T2DM

Current epidemiological data shows approximately 387 million adults worldwide are affected by diabetes, with projections suggesting this number will increase to 693 million by 2045 (Cole and Florez, 2020). Type 2 diabetes (T2DM) is characterized by elevated blood sugar levels, primarily due to impaired β-cell function, leading to inadequate insulin secretion or reduced insulin sensitivity. T2DM affects skeletal muscles through insulin resistance, chronic inflammation, advanced glycation end products, and increased oxidative stress, all of which undermine muscle structure, function, and mass (Chen et al., 2023). This results in muscle loss, decreased quality of life, and a higher risk of disability or death (Kawada, 2021), with diabetics having a 1.5–2 times greater risk of muscle loss than non-diabetics (Izzo et al., 2021). GLUT4, a glucose transporter, manages about 80% of glucose uptake, mainly in skeletal muscles (Son et al., 2017). In T2DM, impaired insulin signaling prevents GLUT4 from effectively transporting glucose, reducing muscle glycogen synthesis and contributing to insulin resistance in muscle tissues. High glucose levels lead to fat accumulation in skeletal muscles, activating protein kinase C-epsilon (PKCε) and worsening insulin resistance (Meex et al., 2019). Increased fat in muscles also releases fatty and inflammatory molecules (Donath and Shoelson, 2011), promoting chronic inflammation, which impairs muscle formation, accelerates muscle breakdown, and causes muscle wasting (Cole and Florez, 2020). Hyperglycemia affects neurons by decreasing their responsiveness to electrical stimuli and altering action potential conduction at the neuromuscular junctions. Additionally, it reduces sarco/endoplasmic reticulum Ca^2+^-ATPase (SERCA) levels (Bayley et al., 2016), impairing skeletal muscle contraction.

### 3.2 NAD^+^ salvage pathways improve the mechanism of skeletal muscle energy metabolism in T2DM

Nicotinamide phosphoribosyltransferase (NAMPT), an adipokine resembling insulin, plays a vital role in the NAD^+^ salvage pathway, which is crucial for the function of pancreatic islet β-cells and is closely linked to glucose and insulin metabolism (Revollo et al., 2007; Yoshino et al., 2011). Research indicates that NMN supplementation can enhance insulin secretion and sensitivity in diabetic mice by improving the NAD^+^ synthesis pathway (Yoshino et al., 2011). Additionally, elevating SIRT1 specifically in the pancreatic islet β-cells of C57BL/6 mice enhances insulin secretion, improves glucose tolerance, and increases insulin sensitivity (Moynihan et al., 2005).NMN supplementation significantly enhances muscle insulin signaling in prediabetic women, improving skeletal muscle insulin sensitivity and increasing glucose uptake and metabolism in skeletal muscle (Yoshino et al., 2021). NR ameliorates insulin resistance in the skeletal muscle of high-fat diet (HFD) mice by activating the AMPK signaling pathway, which inhibits oxidative stress and enhances mitochondrial function (Li et al., 2023). The NAD^+^-dependent enzyme SIRT1 regulates AMPK activity (Imi et al., 2023; Liu and Chang, 2018), which phosphorylates Histone Deacetylase 5 (HDAC5) at Ser259 and Ser498, reducing HDAC5 binding to the GLUT4 gene promoter. This increases GLUT4 gene expression and improves skeletal muscle insulin sensitivity (McGee et al., 2008). AMPK also reduces inflammation and insulin resistance in the skeletal muscle of HFD-fed mice, thereby improving skeletal muscle function in T2DM (Zhao et al., 2018). In the skeletal muscle-specific NAMPT knockout mouse model, k-means clustering analysis was conducted on all detected genes, revealing a distinct cluster associated with inflammation and immune response-related genes (Frederick et al., 2016). The activation of the nuclear factor kappa-B (NF-κB) pathway, a key player in inflammation (van der Heiden et al., 2010), triggers the upregulation of muscle-specific E3 ubiquitin ligase (MuRF1) and cytokines such as TNF, IL-6, and IL-1β. This increases muscle protein breakdown and inhibits protein synthesis, disrupting protein balance and leading to muscle atrophy (Cai et al., 2004). The activity of NF-κB in skeletal muscle of T2DM patients is 2.7 times higher than that of normal glucose tolerant subjects (Tantiwong et al., 2010). Inhibiting NF-κB can significantly reduce muscle loss. Supplementation with NAMPT enhances NAD^+^ synthesis, thereby increasing SIRT1 activity (Stein and Imai, 2012). This activity includes the deacetylation of RelA AcK310 by SIRT1, which inhibits NF-κB transcriptional activity (Yeung et al., 2004), decreases inflammation in skeletal muscles, and improves muscle function in diabetes.

**FIGURE 3:**
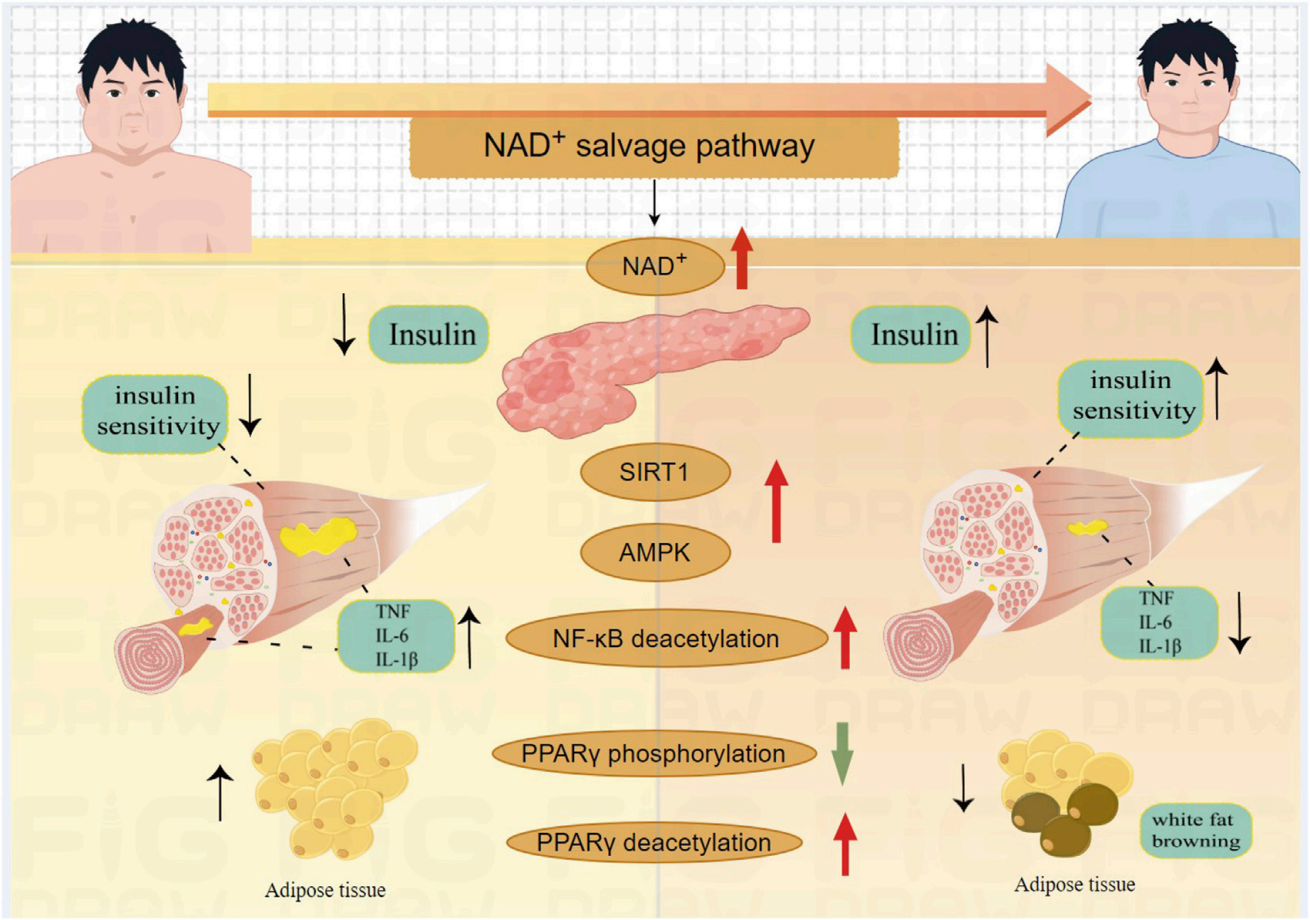
The mechanism of NAD^+^ salvage pathway improve the function of skeletal muscle in T2DM. NAD^+^ enhances insulin secretion; NAD^+^-Sirtuin1- NF-κB decreases inflammation in skeletal muscles; NAD^+^-Sirtuin1-AMPK improves skeletal muscle insulin sensitivity; NAD^+^ decreases PPARγ phosphorylation, promotes fat metabolism; NAD^+^-Sirtuin1 deacetylates PPARγ to induce white fat browning.

Patients with type 2 diabetes and obesity typically exhibit increased fat accumulation around organs, especially in skeletal muscles and liver. Fat accumulation in the skeletal muscle of T2DM patients leads to decreased muscle strength and function (Almurdhi et al., 2016). Adiponectin, a key gene in glucose metabolism (Banks et al., 2015), acts as an insulin sensitizer, increasing insulin sensitivity in skeletal muscle, thereby enhancing glucose uptake and metabolism (Ceddia et al., 2005; Liu et al., 2009). Targeted deletion of NAMPT in mouse adipocytes results in systemic insulin resistance, reduced fat metabolism, lower adiponectin levels, and decreased metabolic efficiency (Stromsdorfer et al., 2016). PPARγ is crucial for fat synthesis, and its complete deletion in mice leads to the loss of both white and brown adipose tissues, causing severe metabolic issues (Gilardi et al., 2019). Additionally, PPARγ suppresses NF-κB activation, which otherwise enhances muscle inflammation (Huang and Glass, 2010). Supplementing PPARγ helps maintain insulin responsiveness in peripheral tissues, increases glucose uptake and utilization in muscles, improves lipid metabolism, and lowers triglyceride levels (Ji et al., 2022). Specific overexpression of NAM in adipose tissue results in a 32-fold increase in NAD^+^ (Luo et al., 2022), levels and a corresponding rise in carnitine content. This enhancement promotes the transport of fatty acids to mitochondria for oxidative breakdown (Rebouche and Seim, 1998). Reduced NAD^+^ levels cause phosphorylation of PPARγ at Ser273 in adipose tissue, which disrupts PPARγ's role in lipid metabolism and inhibits adiponectin function, thereby affecting glucose metabolism (Stromsdorfer et al., 2016). Deacetylating PPARγ at K268 and K293 promotes the recruitment of coactivators that facilitate the browning of white adipose tissue, thereby increasing insulin sensitivity, enhancing energy expenditure, and reducing obesity (Ji et al., 2022). P7C3, an orally active neuroprotective agent, targets the NAMPT enzyme, enhancing the NAD^+^ salvage pathway. This increases insulin sensitivity, boosts mitochondrial fatty acid β-oxidation in skeletal muscle, and improves energy metabolism in T2DM mice (Ravikumar et al., 2022) which is depicted in Figure 3.

## 4 The application of the NAD^+^ salvage pathway in promoting skeletal muscle injury recovery

Rehabilitation and treatment of skeletal muscle injuries remain significant challenges in sports physiology, medicine, and rehabilitation. There is substantial evidence that the NAMPT-mediated NAD^+^ salvage pathway plays a crucial role in the structural and functional recovery of injured skeletal muscles. Studies suggest that extracellular vesicles from young individual’s adipose-derived mesenchymal stem cells (ADMSC young-EVs) are promising for treating tendinopathy by potentially increasing NAMPT expression in tendon cells and macrophages (Wu et al., 2023). Increasing NAMPT expression activates pathways such as NAMPT-SIRT1-PPARγ-PGC-1α, improving mitochondrial functions, reducing cellular damage, and slowing tendon deterioration. Additionally, the activation of the NAMPT-SIRT1-NF-κB/NLRP3 pathway rejuvenates macrophage phagocytic capabilities, lowers pro-inflammatory factor levels, and alleviates tendinopathy. NAMPT enhances M2 macrophage polarization, increasing their proportion, which helps reduce inflammation and promote tendon repair in tendinopathy (Wu et al., 2023). Disuse atrophy occurs when skeletal muscle atrophies due to reduced activity following injury. In models of acute supraspinatus and extensor digitorum longus muscle tears, injecting exogenous NAMPT improves citrate synthase activity and mitochondrial function, thereby enhancing muscle regeneration capabilities (Yao et al., 2023). Dhanushika et al. (2021) discovered in a transgenic zebrafish model that following skeletal muscle injury, about 34% of macrophages quickly migrated to the injury site. A particular subset secretes NAMPT that binds to CCR5 on muscle stem cells (MuSCs), enhancing their repair and proliferation. Research has shown that knocking down SIRT1 disrupts sarcolemma repair and vesicle dynamics at the injury site, increasing vulnerability to further mechanical damage (Fujiwara et al., 2019). Thus, enhancing the NAD^+^ salvage pathway and boosting SIRT1 expression are believed to protect against skeletal muscle damage and hasten sarcolemma recovery.

Exercise is essential for stimulating the regeneration and repair of muscle fibers, and the combination of exercise with NAD^+^ boosters holds promising potential for restoring the function of injured skeletal muscle. Exercise and NAD^+^ boosters may simultaneously enhance the recovery of injured skeletal muscle by improving MuSCs and mitochondrial function. In rodents and humans, exercise and muscle contraction activate AMPK in skeletal muscle (Jørgensen et al., 2006; Winder and Hardie, 1996). Studies have shown that the absence of AMPKα1 leads to a reduction in satellite cell numbers and decreased expression of myogenic factors such as Myf5 and Myogenin (Fu et al., 2015; Thomson, 2018). The AMPKα1-LDH pathway regulates the activation, proliferation, differentiation, and self-renewal of MuSCs (Theret et al., 2017). AMPK enhances sirtuin activity (Cantó et al., 2009; Cantó et al., 2010), and NAD^+^ boosters can also activate sirtuins. High expression of SIRT1 facilitates the recovery of injured muscle function (Myers et al., 2019), while SIRT2 deacetylates PAX7, promoting the self-renewal of MuSCs (Sincennes et al., 2021). AMPK and Sirtuins together can increase PGC-1α activity, which improves mitochondrial function and enhances skeletal muscle regeneration (Wu et al., 2023). The crosstalk between AMPK and Sirtuins offers additional theoretical possibilities for the use of exercise and NAD^+^ boosters in the recovery of injured skeletal muscle. However, the practicality of this approach requires further experimental validation. Furthermore, different types and intensities of exercise also promote NAMPT and NAD^+^ levels, which will be discussed in the next section.

## 5 Exercise regulate NAD^+^ salvage pathway to improve skeletal muscle function strategy

The benefits of regular exercise on skeletal muscles are well-documented. While studies on exercise’s impact on skeletal muscle NAMPT levels are limited, available research indicates that elderly individuals experience a more pronounced increase in NAMPT and NAD^+^ levels from exercise than younger people. Different types of exercise positively affect NAMPT levels, though the specific molecular mechanisms driving these increases are not yet fully understood. Exercise enhances skeletal muscle glycolysis and aerobic metabolism, leading to ATP breakdown into ADP and Pi, releasing energy and producing AMP. This increase in AMP and ADP levels stimulates AMPK phosphorylation at the Thr172 site, enhancing AMPK activity (Hardie et al., 2012). Administering an AMPK activator raises skeletal muscle NAMPT protein levels (Brandauer et al., 2013; Ratia et al., 2023). Furthermore, the level of AMPK activity correlates with exercise intensity, with greater intensity causing more AMPK activation and increased phosphorylation of AMPK (Rothschild et al., 2022). Research suggests that more intense exercise might more effectively elevate NAMPT levels. It is theorized that exercise boosts NAMPT levels primarily through the AMPK-NAMPT pathway, as indicated in Table 1.

**TABLE 1:** Effects of exercises on NAMPT/NAD^+^ in skeletal muscle.

Subjects	Exercise equipment/Mode	Exercise protocol	Tissue sample	NAMPT/NAD^+^ alterations	Reference
Three-month-old male young rats and 26-month-old male aged rats	treadmill	10% incline, 30 min at10 m min^−1^ in 2 weeks; 0% incline, 30 min at 60% VO_2max_ in 3 weeks; 10% incline, 60 min at 22 m/min in 1 week (young rats), and 60 min at 13 m min^−1^ for in 1 week (old rats)	gastrocnemius muscle	Young and aged rats’ NAMPT levels increased, with a marked boost in aged rats, similar to the young controls	Koltai et al. (2010)
13 non-obese sedentary subjects	bicycle	30–60 min at 75%–85% VO_2max_ and 50 min at 70% VO_2max_ for 13x/3 weeks	vastus lateralis	NAMPT increased more than two-fold	Costford et al. (2018)
11 young adults (aged 18–30) and 10 elderly individuals (≥65 years old)	knee extension	In first 4 weeks, 60 min at 65%VO_2max_ for 3x/w; in last 4 weeks 60 min at 65%VO_2max_ for 5x/wk	vastus lateralis	The young showed a significant NAMPT increase, while elderly did not	
8 male minors	knee extension	1 h or 2 h, 15x/3wks	vastus lateralis	Trained group had 16% more NAMPT than untrained	Brandauer et al. (2013)
17 subjects aged 65–80 with a history of exercise for more than 1 year	walk	5 days at 13,671 steps	vastus lateralis	NAD^+^ levels increased, elevating to those of 20–30-year-olds	Janssens et al. (2022)
Forty male participants were grouped based on age (youth, elderly) and VO_2max_ = 45 mL/kg/min as the dividing line	bicycle	20 min at 70%VO_2max_	C2C12 myoblasts	The NAMPT content increased in both the young (VO_2max_ > 45 mL/kg/min) and middle-aged/elderly (VO_2max_ > 45 mL/kg/min) groups, but not in the middle-aged/elderly group with VO_2max_ < 45 mL/kg/min	Chee et al. (2022)
28 wild female mice	treadmill, wheel cages	In 6.5 weeks 1 h at 16 m min^−1^ on weekdays, wheel cages for voluntary running on weekends	quadriceps femoris	NAMPT increased significantly	Brandauer et al. (2013)
21 young adults (aged ≤35) and 22 elderly individuals (aged ≥55)	treadmill, stationary cycle, elliptical trainer	In 12 weeks, 80 min at 70–75%VO_2max_ for 3–4x/wk	vastus lateralis	Youth: 12% NAMPT boost; Elderly: 28% NAMPT surge	De Guia et al. (2019)
16 middle-aged and elderly (59 ± 4 years old) untrained subjects	leg/hip sled, lying leg curls, leg extensions, barbell bench press, cable pull downs	In 10 weeks, 70% max intensity for 10–12*3groups, 1 min interval for 2x/wk	quadriceps femoris	NAD+ and NAMPT up 127% and 115%, respectively, NAD^+^ like 22 ± 3 year old	Lamb et al. (2020)
21 young adults (aged ≤35) and 22 middle-aged/elderly individuals (aged ≥55)	dumbbell, barbell	In 12 weeks, 45 min/x for 3/w. when 12 repetitions was completed on two consecutive occasions, resistance increased by 5%	vastus lateralis	The NAMPT levels in young adults and middle-aged/elderly individuals increased by 25% and 30%, respectively	De Guia et al. (2019)
32 male mice	treadmill	10 min at 13 m min^−1^, 80 min at 17 m min^−1^	quadriceps femoris	NAMPT mRNA expression increased 3 h later	Brandauer et al. (2013)

## 6 NAD^+^ boosters

For individuals unable to perform physical exercise due to injury or chronic disease, NAD^+^ boosters might serve as exercise mimetics, activating similar biochemical pathways that are engaged during physical exercise, thus maintaining muscle function and promoting metabolic health. Modulators of enzymes of the NAD^+^ biosynthesis pathway, NAD^+^ precursors and NAD^+^ consuming enzyme inhibitors are main NAD^+^ boosters. We will discuss the prospects, side effects, and current status of clinical transformation of NAMPT agonists, NAD^+^ precursors and NAD^+^-consuming enzyme inhibitors to provide a more balanced and unbiased perspective on NAD^+^ metabolism.

### 6.1 NAMPT agonists

NAMPT agonists can enhance the efficiency of the body’s salvage synthesis pathway to produce NMN, and even with small or intermittent supplementation of NMN or NR, they can maintain high levels of NAD^+^ in the long term. Therefore, many studies have attempted to explore the types and potential roles of NAMPT agonists. P7C3 is the first discovered activator of NAMPT. The active variants of P7C3 can enhance the activity of purified NAMPT, and their promotion of NMN production is dose-dependent (Wang et al., 2014). Research has shown that P7C3-mediated NAMPT activation improves insulin sensitivity and muscle function in obese mice, reducing diabetic symptoms (Ravikumar et al., 2022). The exact mechanisms of how P7C3 activates NAMPT or competes with its inhibitors are still under investigation. The synthesis of SBI-797812, an NAMPT activator, involved modifying the GNI-50 molecule by moving the pyridine group from the third to the fourth position. This change enhances NAMPT’s catalytic efficiency, increasing NMN synthesis by 2.1 times (Gardell et al., 2019). SBI-797812 also counteracts the inhibitory effects of NAD^+^ on NAMPT by binding to its rear channel, boosting NAMPT activity and increasing NAD^+^ levels (Ratia et al., 2023). NAT and its active variant NAT-5r, potent NAMPT activators, interact through a hydrogen bond with NAMPT’s K189 residue, enhancing its catalysis and countering FK866-induced cell death (Hong et al., 2022). High-throughput screening has identified several compounds that enhance NAMPT function, including NAMPT positive allosteric modulators A1 (NP-A1) (Zhengnan et al., 2023). These modulators increase NAMPT activity by 1.6–2.6 times, binding to its back channel (Ratia et al., 2023). N-PAMs reduce NAD^+^'s inhibitory effect on NAMPT, enhancing its function. Natural activators like notoginseng leaf triterpenes (PNGL) also stimulate NAMPT (Xie et al., 2020), activating NAMPT-NAD^+^-SIRT1/2/3-Foxo3a-MnSOD/PGC-1α pathways that improve mitochondrial function and prevent mitochondrial damage. The hypertension peptide IRW increases muscle cell NAMPT content significantly (Bhullar et al., 2021). Additionally, substances like low-dose nicotine enhance NAMPT activity by facilitating its interaction with SIRT1, boosting NAD^+^ and β-NMN levels, and improving metabolic function in aging tissues (Liang et al., 2023).

Although NAMPT agonists have shown therapeutic potential in laboratory studies, they have revealed certain safety concerns in clinical trials, their therapeutic effects do not yet meet clinical needs, and the progress of clinical trials has been slow (Wen et al., 2024). Moreover, NAMPT regulates macrophage survival and pro-inflammatory activity, contributing to the modulation of the tumor inflammatory microenvironment and promoting tumor cell metastasis (Lucena-Cacace et al., 2018; Piacente et al., 2017). NAMPT activity appears to be tightly controlled by NMN and NAD^+^ through strict feedback regulation (Burgos and Schramm, 2008; Takahashi et al., 2010). Developing NAMPT agonists that can specifically enhance NAD^+^ production without affecting other functions, such as immune response regulation and cancer cell metabolism, remains a significant challenge.

### 6.2 NAD^+^ precursors

NAD^+^ precursors, such as NA, NMN, NR, and NAM, have gained significant attention for NAD^+^ supplementation and have been shown in both animal and human studies to extend lifespan, enhance muscle regeneration, improve mitochondrial and stem cell function, boost glucose metabolism, and enhance cardiovascular function. The specific effects of these precursors on the body are detailed in several excellent reviews (Iqbal and Nakagawa, 2024; Keisuke and Takashi, 2023; Lautrup et al., 2024; Montllor-Albalate et al., 2021; Reiten et al., 2021). However, different NAD^+^ precursors exhibit varying effects depending on the experimental subjects. For instance, postmenopausal overweight or obese women with prediabetes who took NMN(250 mg/day) for 10 weeks showed significant improvements in muscle insulin sensitivity (Yoshino et al., 2021). In contrast, healthy, obese, sedentary men aged 40–70 who received NR (2000 mg/day) for 12 weeks did not experience improvements in insulin sensitivity, endogenous glucose production, glucose handling, or oxidation (Dollerup et al., 2018). Older men with diabetes and impaired physical function did not see improvements in muscle strength after taking NMN 250 (mg/day) for 24 weeks (Akasaka et al., 2023). Patients with mitochondrial myopathy who took NA 750–1,000 (mg/day) for four months showed improvements in muscle NAD^+^ levels, disease symptoms, and muscle metabolism (Pirinen et al., 2020). These variations in outcomes may be associated with factors such as gut microbiota, dose dependency, and individual differences. Currently, NAD^+^ precursors such as NA, NAM, NR, and NMN are used clinically and have demonstrated certain levels of safety and bioavailability (Reiten et al., 2021). However, more comprehensive research is needed to evaluate their potential side effects and therapeutic efficacy. The interactions of gut microbiota with NAD^+^ precursors add complexity to NAD^+^ metabolism. Optimizing precursor formulations and administration methods could enhance their stability and conversion efficiency in the body. Furthermore, exploring targeted delivery methods of NAD^+^ precursors to specific organs or tissues could improve clinical trial outcomes.

### 6.3 NAD^+^-consuming enzyme inhibitors

NAD^+^ boosters also include inhibitors of NAD^+^-consuming enzymes. CD38 inhibitors improve age-related metabolic disorders by reversing tissue NAD^+^ decline, increasing muscle fiber size, and reducing fibrosis (Tarragó et al., 2018). CD38-deficient mice prevent high-fat diet-induced obesity by activating the SIRT-PGC1α axis through elevated NAD^+^ levels (Barbosa et al., 2007). The deletion of the PARP gene increases NAD^+^ content and SIRT1 activity in brown adipose tissue and muscle, enhancing mitochondrial content and oxidative metabolism (Bai et al., 2011). However, using PARP inhibitors could have adverse effects, as PARP is involved in essential cellular processes, and its inhibition may lead to genomic instability (Beneke et al., 2004). The development of NAD^+^ consumption enzyme inhibitors requires comprehensive consideration of drug safety, its impact on tumor development, the role of inflammation and metabolic regulation, biological rhythms and adaptive responses, individual differences, and optimization of supplementation strategies. These challenges need to be fully addressed in future research.

NAMPT agonists accelerate NAD^+^ synthesis, NAD^+^ precursors provide direct supplementation, and consumption inhibitors reduce NAD^+^ degradation, forming a multifaceted synthesis and protection network that significantly enhances intracellular NAD^+^ levels. This synergistic approach holds promise as a target for treating diseases and disorders associated with disrupted NAD^+^ homeostasis.

## 7 Conclusion

In skeletal muscle, efficient energy metabolism is crucial, and disruptions in NAMPT and NAD^+^ levels or synthesis significantly impact muscle function. These disturbances are particularly evident in conditions like aging, type 2 diabetes (T2DM), and muscle injuries, where NAMPT-mediated NAD^+^ salvage pathways are compromised. The NAD^+^ salvage pathway improves function in aging skeletal muscles by reducing oxidative stress, stabilizing mitochondrial NAD^+^ pools, promoting autophagy, reducing chronic low-grade inflammation, restoring neuromuscular junction (NMJ) functionality, and enhancing MuSCs quantity and function. Similarly in T2DM, it enhances muscle function by improving insulin secretion, enhancing skeletal muscle insulin sensitivity, reducing inflammation, increasing fatty acid oxidation, and promoting the browning of white adipose tissue. In muscle injury scenarios, the NAD^+^ salvage pathway facilitates MuSCs proliferation and mitigates mitochondrial dysfunction, thereby enhancing muscle contractility. Exercise similarly exploits this pathway to bolster skeletal muscle functionality. Therefore, manipulating the NAD^+^ salvage pathway holds significant potential for enhancing skeletal muscle function. NAD^+^ boosters, in particular, could be effective in treating various muscle dysfunctions and enhancing overall muscle health.
